# Machine‐learning derived proxy phenotypes for Alzheimer’s disease genome‐wide association study by proxy

**DOI:** 10.1002/alz.094126

**Published:** 2025-01-09

**Authors:** Benjamin Goudey, Martin Saint‐Jalmes, Shu Liu, Simon M. Laws, Colin L Masters

**Affiliations:** ^1^ Florey Institute of Neuroscience and Mental Health, University of Melbourne, Parkville, VIC Australia; ^2^ ARC Training Centre in Cognitive Computing for Medical Technologies, Parkville, VIC Australia; ^3^ Collaborative Genomics and Translation Group, School of Medical and Health Sciences, Edith Cowan University, Joondalup, Western Australia Australia

## Abstract

**Background:**

For large genomic studies of middle‐aged individuals, the prevalence of Alzheimer’s disease (AD) is extremely low, making it difficult to conduct genomic analysis of the condition. To enable genome‐wide association studies of AD in such datasets, an approach called Genome‐wide association by proxy (GWAX) uses family history of disease as a proxy for disease status. Borrowing from the machine learning (ML) literature, we treat the development of proxy phenotypes as a pseudo‐labelling task, where an ideal proxy label accurately predicts the lifetime risk of AD. Given this, this work develops ML‐derived proxy phenotypes of AD for downstream GWAX based on survival models of conversion to AD and demonstrates their predictive performance and impact on downstream GWAX.

**Method:**

Using cognitively normal (CN) and mild cognitive impaired (MCI) individuals with at least 2 timepoints in AIBL (n = 426), ADNI (n = 283) and UK Biobank (n = 56,105), we derived two proxy phenotypes of AD: i) a ML‐derived boosting model with age, sex, years of education and AD family history, i.e. number of recorded parents with a history of Alzheimer’s disease and ii) family history alone. The prognostic ability for future AD conversion was measured using concordance index (C‐index) in 10‐fold cross‐validation. The two models were applied to the subset of UK Biobank with white British ancestry (n = 408,165) and a GWAX was conducted on the predicted labels. From this GWAX, PRS was derived and evaluated using area under the ROC curve (AUC).

**Results:**

In 10‐fold cross‐validation, the ML‐derived phenotypes were more predictive of conversion to AD than family history alone (median C‐index: 0.7 vs 0.5, Mann Whitney p‐value <0.001). GWAX of UKB from these proxy phenotypes indicates that the multivariate model detects a higher number of significant regions compared to family history alone. PRS constructed from these ML‐derived phenotypes GWAX show greater power to separate true AD case/control labels when externally validated on ADNI.

**Conclusion:**

Preliminary analysis of ML‐derived proxy phenotypes of AD indicates may be a promising approach to improve AD genomic studies in middle age cohorts. Further analysis of the results and potential biases of this approach are needed to fully characterise this method.

